# Determination of Imidazole Dipeptides and Related Amino Acids in Natural Seafoods by Liquid Chromatography–Tandem Mass Spectrometry Using a Pre-Column Derivatization Reagent

**DOI:** 10.3390/foods13121951

**Published:** 2024-06-20

**Authors:** Mayu Onozato, Minori Horinouchi, Yuki Yoshiba, Tatsuya Sakamoto, Hiroshi Sugasawa, Takeshi Fukushima

**Affiliations:** Department of Analytical Chemistry, Faculty of Pharmaceutical Sciences, Toho University, 2-2-1 Miyama, Funabashi-shi 274-8510, Chiba, Japan; mayu.onozato@phar.toho-u.ac.jp (M.O.); 1020192h@st.toho-u.jp (M.H.); 1020237y@st.toho-u.jp (Y.Y.); tatsuya.sakamoto@phar.toho-u.ac.jp (T.S.); 3022006s@st.toho-u.jp (H.S.)

**Keywords:** imidazole dipeptide, carnosine, anserine, alanine, histidine, taurine, chiral derivatization, LC–MS/MS

## Abstract

Imidazole dipeptides (IDPs) and taurine (Tau) have several health benefits and are known to be contained in natural seafoods. However, their levels vary widely in different natural seafoods, making their simultaneous determination desirable. Herein, we employ a liquid chromatography–tandem mass spectrometry approach using a novel amino group derivatization reagent, succinimidyl 2-(3-((benzyloxy)carbonyl)-1-methyl-5-oxoimidazolidin-4-yl) acetate ((*R*)-CIMa-OSu), for the simultaneous quantification of IDPs (carnosine (Car) and anserine (Ans)), their related amino acids, and Tau in natural seafoods. Each seafood sample contained different concentrations of IDPs (Car: ND to 1.48 mmol/100 g-wet, Ans: ND to 4.67 mmol/100 g-wet). The Car levels were considerably higher in eel, while Tau was more abundant in squid, boiled octopus, and scallop. Thus, the derivatization reagent (*R*)-CIMa-OSu provides a new approach to accurately assess the nutritional composition of seafoods, thereby providing valuable insight into its dietary benefits.

## 1. Introduction

**Figure 1:**
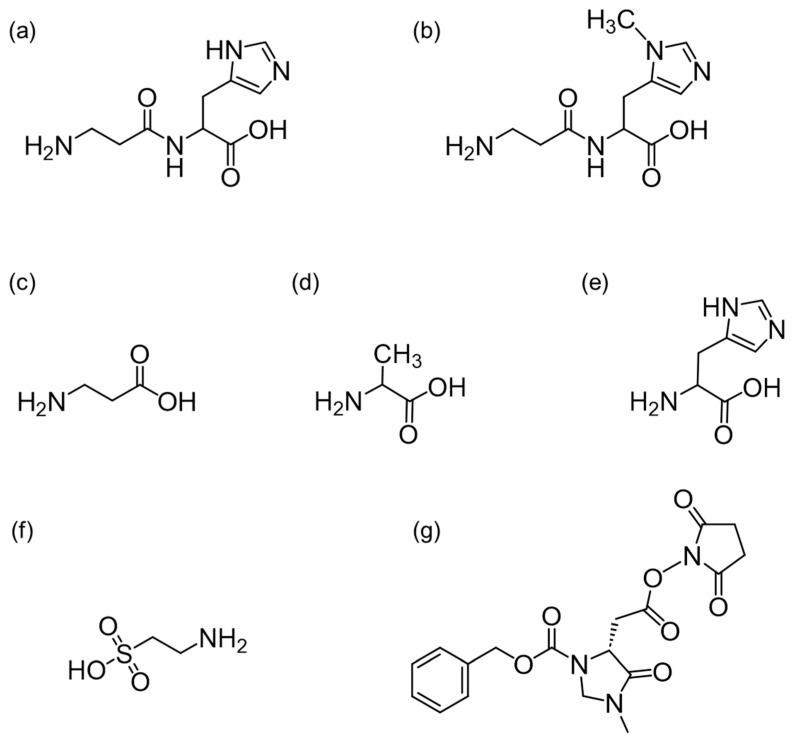
Chemical structures of (**a**) carnosine (Car), (**b**) anserine (Ans), (**c**) β-alanine (β-Ala), (**d**) alanine (Ala), (**e**) histidine (His), (**f**) taurine (Tau), and (**g**) succinimidyl 2-(3-((benzyloxy)carbonyl)-1-methyl-5-oxoimidazolidin-4-yl) acetate ((*R*)-CIMa-OSu).

Carnosine (Car, Figure 1a) and anserine (Ans, Figure 1b) are well-known imidazole dipeptides (IDPs). Car comprises β-alanine (β-Ala, Figure 1c), a structural isomer of alanine (Ala, Figure 1d), and histidine (His, Figure 1e), while Ans comprises β-Ala and 1-methylhistidine. Car and Ans are found in the skeletal muscles of mammals (e.g., cows, pigs, and chicken) and fish (e.g., eel and tuna) [1]; therefore, they can be consumed through food.

Car has various interesting properties, including antioxidant [2], metal chelating [3], anti-glycation [4], anti-inflammatory [5], and anti-aging activities [6]. Additionally, due to its pH buffering activity [7], Car may be involved in decreasing muscle fatigue during exercise. It also has positive effects on cognitive recovery and brain function (500 mg Car/Ans (1:3) twice daily [8] or 1 g Car/Ans (1:2) once daily [9]). Consequently, various supplements containing Car have been commercialized. Ans possesses functions similar to those of Car, including good antioxidant activity [10,11], suppression of inflammatory cytokine production [12], and enhancement of muscle strength and endurance [13]. Additionally, the effects of Ans on glycemia [14] and uric acid levels [15] have been reported.

Following consumption, Car is absorbed by the small intestine via peptide transporter-1 (Pep T1). A small amount of Car is hydrolyzed to β-Ala and His by carnosinase-2 (CN-2, EC 3.4.13.18) in the enterocytes, while the majority passes through the basolateral membranes of the intestinal cells and enters the portal circulation [16], where it is hydrolyzed to β-Ala and His by carnosinase-1 (CN-1, EC 3.4.13.20) [17]. Car has been detected in human tissues, including the skeletal muscles, central nervous system, heart, kidneys, adipose tissue, lungs, and liver. The expression of Car synthase (CARNS 1, EC 6.3.2.11) has been identified in three sites (i.e., skeletal muscles, olfactory bulbs, and the central nervous system), wherein it is resynthesized from β-Ala and His [18]. Because Car is degraded by CN-1 and CN-2, it is hardly detected in blood, but it is detected in muscle.

Ans was initially not detected in human muscle due to its low concentration and the low sensitivities of the measurement instruments. However, with various advancements in analytical techniques, Hoetker et al. detected Ans in human skeletal muscle [19], and subsequently, Goncalves et al. reported the detection of Ans in both the cardiac and skeletal muscles of humans [20]. The expression of Car *N*-methyltransferase 1 (CARNMT-1, EC 2.1.1.22) has also been confirmed in human skeletal muscles, indicating that the His component of Car is methylated to produce Ans [20].

Taurine (Tau, Figure 1f) also plays an important role in several biological processes, such as membrane stabilization, osmotic regulation, and the development of the central nervous system and retina, in addition to exhibiting antioxidant effects [21]. Fish and shellfish, especially invertebrates such as mollusks and crustaceans, reportedly contain high concentrations of Tau [22]. It is therefore desirable to determine the contents of IDPs and Tau in natural seafoods. To our knowledge, few studies have examined the variations in the IDP and Tau contents of different natural seafoods.

High-performance liquid chromatography (HPLC) coupled with fluorescence detection or mass spectrometry (MS) is highly efficient for simultaneously analyzing amino acids and dipeptides. For fluorescence detection, various pre-column derivatization reagents have been employed, including dansyl chloride, 2,5-dioxopyrrolidin-1-yl *N*-tri(pyrrolidino)phosphoranylideneamino carbamate, and 9-fluorenylmethyl chloroformate. Similarly, *p*-*N*,*N*,*N*-trimethylammonioanilyl *N*’-hydroxysuccinimidyl carbamate iodide and diethyl ethoxymethylenemalonate have been used in MS [23,24]. Our laboratory previously developed succinimidyl 2-(3-((benzyloxy)carbonyl)-1-methyl-5-oxoimidazolidin-4-yl) acetate ((*R*)-CIMa-OSu, Figure 1g) for application in MS [25]. Notably, (*R*)-CIMa-OSu has an asymmetric carbon atom, which facilitates the enantiomeric separation of amino acids. Consequently, this derivatization reagent was successfully applied to determine not only the l-amino acid, but also the d-amino acid contents in various fermented foods, including miso [25] and black garlic [26]. In addition, its applicability was extended to the analysis of d-amino acids in aquatic invertebrates, specifically polychaetes [27], revealing large concentration differences across polychaete species.

In the current study, (*R*)-CIMa-OSu was employed to quantify Car, Ans, and Tau in several natural seafoods via liquid chromatography–tandem mass spectrometry (LC MS/MS). The related amino acids (d- and l-His and d-, l-, and β-Ala) were also quantified. The proposed technique enables the simultaneous determination of IDPs and the related amino acids. Subsequently, the levels of these components in natural seafoods were compared and evaluated. The use of (*R*)-CIMa-OSu offers several advantages over previous derivatization reagents, including enantiomeric separation, higher detection sensitivity, and the ability to analyze multiple components simultaneously. These features make it a powerful tool for the comprehensive analysis of amino acids and peptides in complex biological samples.

## 2. Materials and Methods

### 2.1. Chemicals and Reagents

l-Ala and l-His were obtained from Kyowa Hakko Bio (Tokyo, Japan). l-Car, d-Ala, Tau, HPLC-grade CH_3_OH (MeOH), LC–MS-grade MeOH, HPLC-grade formic acid, and the APDSTAG^®^ Wako Amino Acids Internal Standard Mixture Solution were obtained from FUJIFILM Wako Pure Chemical Co., Ltd. (Osaka, Japan); 4-Dimethylaminopyridine (DMAP), triphenylphosphine, 2,2′-dipyridyl disulfide, β-Ala, and d-His were purchased from Tokyo Chemical Industry (Tokyo, Japan). HPLC-grade CH_3_CN and LC–MS-grade CH_3_CN were obtained from Kanto Kagaku (Tokyo, Japan). Phosphate-buffered saline (PBS) was purchased from Nissui (Tokyo, Japan). l-Ans was purchased from MedChemExpress Co., Ltd. (Monmouth Junction, NJ, USA). The water used in all experiments was purified using a Milli-Q Lab system (Nihon Millipore, Tokyo, Japan). Millex^®^-LG filters (0.20 μm) were purchased from Merck (Darmstadt, Germany).

### 2.2. Reaction of IDPs and Amino Acids with (R)-CIMa-OSu

A mixed standard solution (200 μM each of β-Ala, d-Ala, l-Ala, l-His, d-His, Tau, Car, and Ans, 10 μL), 20 mM (*R*)-CIMa-OSu in CH_3_CN (10 μL), and 30 mM DMAP in CH_3_CN (10 μL) were vortexed for 1 min then allowed to react for 60 min at 22 °C. Subsequently, 0.1% formic acid in CH_3_CN (70 μL) was added to the reaction solution along with the mobile phase (A:B = 9:1 *v*/*v*, 100 μL, as defined in Section 2.3).

### 2.3. LC–MS/MS Analysis

The LC–MS/MS instrument, mobile phase, column, mobile phase composition, and mobile phase gradient were reported previously [26,27]. Briefly, a triple quadrupole LCMS-8040 mass spectrometer (Shimadzu, Kyoto, Japan) attached to an electrospray ionization interface was used, along with two pumps (LC-20AD), an autosampler (SIL-20AC), a column oven (CTO-20A), and PC software (LabSolutions ver. 5.80, Shimadzu). The temperature of the autosampler tray was set at 4 °C. The analytical column was a Scherzo SS-C18^®^ column (250 × 2.0 mm; i.d., 3 μm; Imtakt, Kyoto, Japan) maintained at 60 °C in the column oven. The mobile phase, which consisted of H_2_O/MeOH/10 mM ammonium formate in H_2_O (pH 2.8) (50:20:30 *v*/*v*/*v*) (solvent A) and 10 mM ammonium formate in H_2_O/MeOH (30:70 *v*/*v*) (solvent B), was pumped constantly at a flow rate of 0.2 mL/min using the following elution program: 0–20 min, 10% B; 20.01–56 min, 10–59% B; 56.01–60 min, 59–100% B; 60.01–75 min, 100% B; 75.01–90 min, 10% B. The injection volume was 5.0 μL. The desolvation line and heatblock temperatures were adjusted to 250 and 400 °C, respectively. The flow rates of the nebulizing and drying gases, the ion spray voltage, and the collision-induced dissociation (CID) gas pressure were 3.0 and 10 L/min, 4.5 kV, and 230 kPa, respectively.

The fragmentation patterns of the IDPs and amino acids coupled with (*R*)-CIMa-OSu were measured in negative ion mode under four collision energies (CEs; 10, 20, 30, and 40 V), and the clearest mass spectra were obtained at the optimal CE for each IDP and amino acid. The optimal CEs were as follows: 10 V for l- and β-Ala; 20 V for Car, Ans, and l-His, and 30 V for Tau. Subsequently, the intensity of the *m*/*z* 91.1 signal was measured in positive ion mode at different CEs (−5 to −90 V).

Each 10 mM standard solution was individually derivatized with 20 mM (*R*)-CIMa-OSu, as described in Section 2.2. The derivatized IDPs and amino acids were individually injected directly into the mass spectrometer to optimize the compound-dependent parameters and obtain the multiple reaction monitoring (MRM) conditions (Table S1). These MRM conditions were employed for sample quantification.

### 2.4. Sampling of Fish and Shellfish

Cultured eels (~200 g) were purchased from a local supplier, CHUHEI (Choshi-shi, Chiba, Japan) in October 2023. Nine types of sashimi (tuna, skipjack tuna, salmon, red seabream, yellowtail, horse mackerel, sardine, squid, and scallops) and boiled octopus were purchased from four local stores (Ichikawa-shi, Chiba, and Koto-ku, Tokyo, Japan) between January and February 2024. All samples were purchased as foodstuffs; that is, in the prepared state and intended for human consumption. All samples (except the boiled octopus) were in the fresh and raw state. The sashimi were sliced in fillets. The eel was whole and the boiled octopus was only a leg. Each slice of sashimi weighed approximately 8–13 g (Table S2). The skipjack tuna and sardine sashimi were slightly larger than the other sashimi, while the squid sashimi was slightly lighter because it was cut into thin strips. There were no notable differences in weight among purchasing locations.

Homogenates were prepared by cutting pieces from the edible parts of each seafood (200 mg each, *n* = 3 per seafood; each piece was from a different fillet slice, eel, or octopus). The pieces were then placed in 5.0 mL tubes along with ice-cold PBS (1.0 mL per 100 mg sample), followed by homogenization using a ShakeMan 6 instrument (Biomedical Science, Tokyo, Japan) with two 6 mm stainless steel beads. Homogenization was conducted for 30 s at 4350 rpm followed by a 5 s rest, then repeated at least three times. Subsequently, the resulting suspension was centrifuged at 13,200× *g* and 4 °C for 15 min. The obtained supernatant was then transferred to a 1.5 mL tube, diluted five-fold using ice-cold PBS, and stored at −80 °C.

### 2.5. Derivatization of IDPs and Amino Acids in Fish and Shellfish Samples

The homogenates (10 μL) were thawed as required and then mixed with internal standard (IS) (ASPDTAG^®^ Wako Amino Acids IS mix, 10 μL), H_2_O (10 μL), and MeOH/CH_3_CN (1:1 *v*/*v*, 120 μL), followed by vortexing for 1 min. After centrifugation (13,200× *g*, 4 °C, 5 min), the supernatant (120 μL) was transferred to a 1.5 mL brown tube and evaporated at room temperature until the sample was completely dry. Water (10 μL) was then added to the dried residue and vortexed for 1 min, followed by the addition of 20 mM (*R*)-CIMa-OSu in CH_3_CN (10 μL) and 30 mM DMAP in CH_3_CN (10 μL). The solution was vortexed for a further 1 min and allowed to stand at room temperature (22 °C) for 60 min. Subsequently, a 0.1% solution of formic acid in CH_3_CN (70 μL) was added to stop the derivatization reaction. The mobile phase (A:B = 9:1 *v*/*v*, 100 μL, as defined in Section 2.3) was then added. After vortexing for a further 1 min, the solution was filtered using a Millex^®^-LG filter (0.20 μm) and analyzed by LC–MS/MS.

The (*R*)-CIMa-OSu derivatives of the IDPs and amino acids in the seafood specimens were detected using the MRM mode ([M + H]^+^ > 91.1, as described in Section 2.3 and Table S1) [26]. The *m*/*z* 91.1 ion is a benzyl cation and the most abundant product ion detected in positive ion mode for all CIMa derivatives. For individual seafood analysis, samples were prepared in duplicate, and the mean value was taken as the concentration. The concentrations of IDPs and amino acids in the seafood samples were then calculated as the mean of the component concentrations (mmol/100 g-wet) detected for three pieces (mean ± standard deviation, SD). When the same kind of seafood was purchased from multiple stores, the data were combined. The concentrations of IDPs and amino acids were then compared between samples.

### 2.6. Validation

The calibration curves for the IDPs and amino acids were prepared by plotting the sample/IS peak area ratio against the concentration (*n* = 4). The concentrations were set according to the individual contents in the homogenates. Specifically, the minimum concentration was typically 6.25 μM, and the maximum concentrations were 200 μM for β-Ala, d-Ala, and d-His, 500 μM for Car, 1000 μM for l-Ala and Tau, 2000 μM for Ans, and 3000 μM for l-His. All concentration levels were set using at least six points (Table S3).

The intra- and inter-assay accuracies and precisions of the LC–MS/MS method were evaluated by spiking three concentrations of standard solutions into the eel homogenate (*n* = 4, Table S4). Thereafter, the IS was added to this mixed sample and pre-treated in the same way as the fish samples (see Section 2.5), and the IDPs and amino acids were quantified (see Section 2.5). The limits of detection (LODs) were calculated based on a signal-to-noise ratio of 3 (S/N = 3).

## 3. Results and Discussion

### 3.1. Mass Spectra of the (R)-CIMa-OSu Derivatives of Car, Ans, His, β-Ala, and Tau

**Figure 2:**
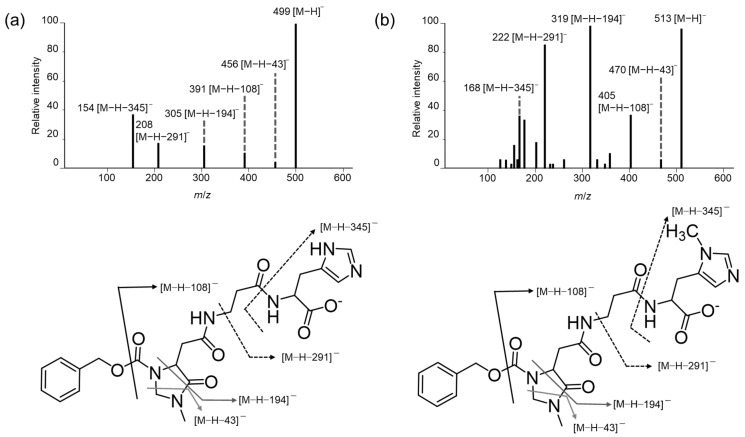
Negative collision-induced dissociation (CID) mass spectra and proposed mass fragmentation patterns of (**a**) CIMa-Car and (**b**) CIMa-Ans.

**Figure 3:**
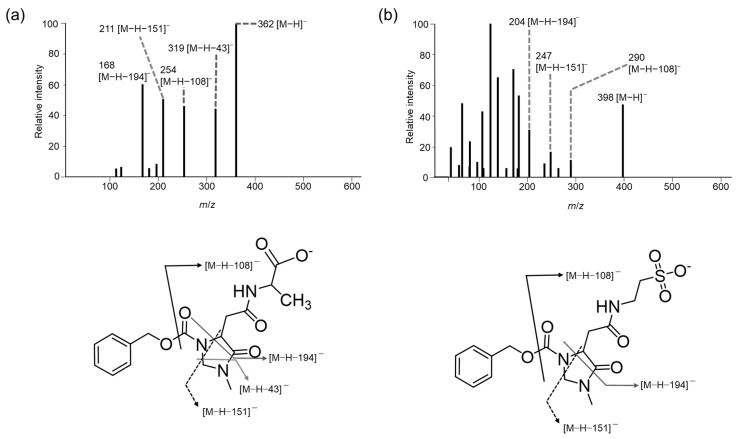
Negative CID mass spectra and proposed mass fragmentation patterns of (**a**) CIMa-Ala and (**b**) CIMa-Tau.

The primary amino groups of Car, Ans, His, β-Ala, and Tau were derivatized using (*R*)-CIMa-OSu, and the produced derivatives were separated and detected by LC–MS/MS. In negative ion mode, ion fragments [M − H − 43]^−^, [M − H − 108]^−^, and [M − H − 194]^−^ were observed for the IDP derivatives CIMa-Car (Figure 2a) and CIMa-Ans (Figure 2b), as well as the amino acid derivatives CIMa-Ala (Figure 3a), CIMa-β-Ala (Figure S1a), and CIMa-His (Figure S1b). Meanwhile, the [M − H − 151]^−^ fragment was uniquely detected for the amino acid derivatives, whereas [M − H − 291]^−^ and [M − H − 345]^−^ were uniquely identified for CIMa-Car and CIMa-Ans. The [M − H − 291]^−^ fragment has not been detected for IDPs previously; therefore, it may be useful for identifying IDPs and compounds possessing previously unidentified amino groups in non-targeted analyses using (*R*)-CIMa-OSu in the future. Although the [M − H − 345]^−^ fragment was also only detected for the IDP derivatives, it has been detected previously for non-derivatized IDPs [28,29,30].

The [M − H − 43]^−^ fragment was not observed for the Tau derivative CIMa-Tau in negative ion mode (Figure 3b). For the CIMa-Ala derivative (Figure 3a), this fragment is produced as follows. First, the carboxyl group of Ala is ionized by the negative ion, producing a carboxylate anion. This anion transfers its negative charge to the nitrogen in the amine structure of CIMa. The negative charge then attacks the carbonyl group of the imidazolidine ring, which eliminates CH_2_NCH_3_ and produces an imide ion ([M − H − 43]^−^) [25]. However, since CIMa-Tau has a sulfonic acid group instead of a carboxyl group, this attack and elimination fragmentation does not occur. A similar trend was observed with another amino group derivatization reagent developed in our laboratory, Ns-MOK-(*S*)-β-Pro-OSu [31].

**Figure 4:**
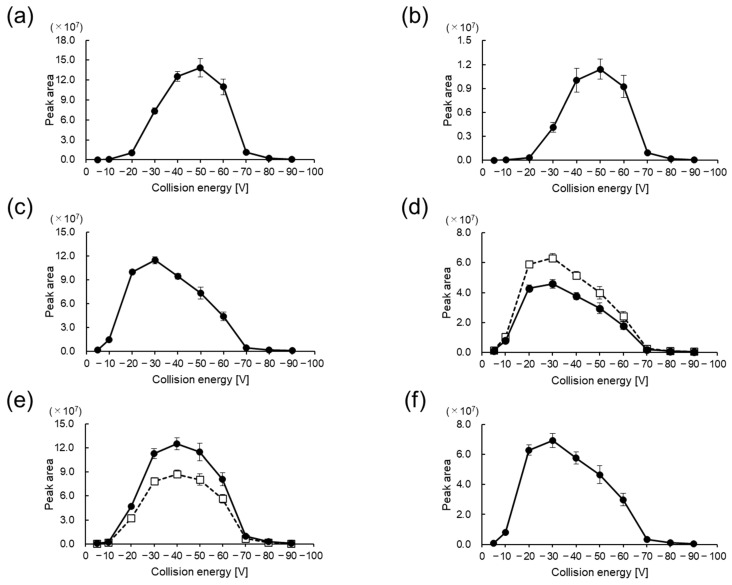
Peak area profiles of benzyl cations (*m*/*z* 91) with variation in the collision energy (CE) in positive ion mode for CIMa-OSu derivatives of (**a**) Car, (**b**) Ans, (**c**) β-Ala, (**d**) l-Ala (solid line with filled circles) and d-Ala (dashed line with open squares), (**e**) l-His (solid line with filled circles) and d-His (dashed line with open squares), and (**f**) Tau.

In positive ion mode, the production of the *m*/*z* 91 fragment, which has previously been used to quantify amino acids in foods [26] and aquatic biological samples [27], was found to depend on the CE (Figure 4). The CEs that most frequently produced the *m*/*z* 91 ion were −30 V for CIMa-Ala (Figure 4d) and CIMa-Tau (Figure 4f), −40 V for CIMa-His (Figure 4e), and −50 V for CIMa-Car (Figure 4a) and CIMa-Ans (Figure 4b). Considering the trend of optimal CE values obtained in this study, it was found that as the number of amino groups to be derivatized increases, a larger CE is required when using (*R*)-CIMa-OSu for non-targeted analysis. Therefore, the relationships among the CE, fragments, and their intensities should be carefully investigated.

### 3.2. Separation of Car, Ans, His, β-Ala, and Tau Derivatives

A mixed-mode stationary phase (i.e., Scherzo^®^ SS-C18) was employed for analysis of the (*R*)-CIMa-OSu derivatives of Car, Ans, His, β-Ala, and Tau. This stationary phase was adopted because it facilitated the enantiomeric separation of amino acids in our previous study [26]. Mixed-mode columns are useful for analytes with ionic groups, such as carboxyl and amino groups, because they allow hydrophobic and ion exchange interactions to occur simultaneously [26]. Consequently, CIMa-His was enantiomerically separated into CIMa-d-His and CIMa-l-His. However, the enantiomeric separation of CIMa-Car into CIMa-d-Car and CIMa-l-Car failed, with only a single peak being eluted. The failure to separate CIMa-Car with the current derivatization reagent may be because the distance between the reaction site of CIMa-OSu and the chiral carbon in Car is larger than that for amino acids. These results suggest that the separation of Car enantiomers by pre-column diastereomer derivatization is particularly challenging, likely due to the presence of a flexible ethylene moiety between the two chiral moieties, (*R*)-CIMa and His. An alternative approach, such as the use of a chiral stationary phase, may therefore be necessary [32].

### 3.3. Validation Data for Car, Ans, His, β-Ala, and Tau Derivatives

**Figure 5:**
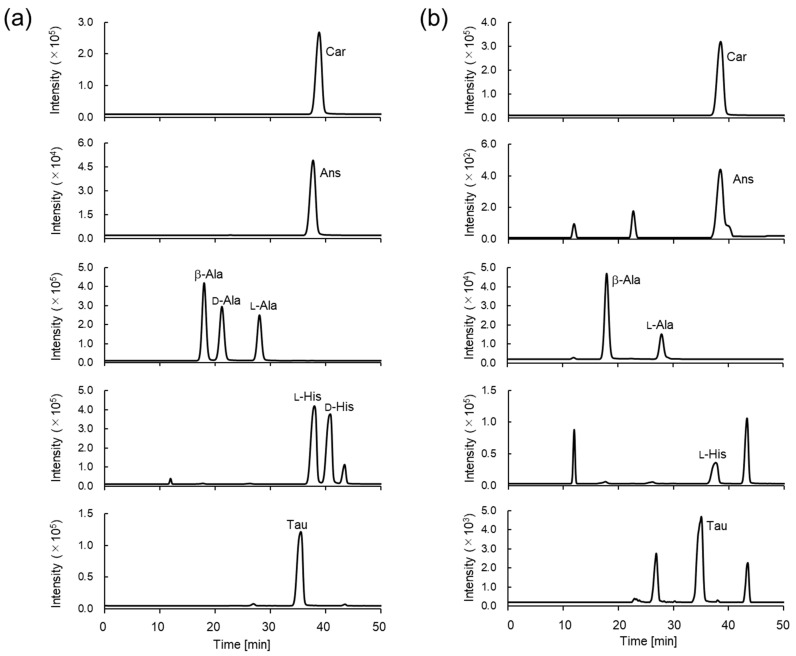
Representative multiple reaction monitoring (MRM) chromatograms of derivatized imidazole dipeptides (IDPs) and amino acids in (**a**) standard solutions (200 μM each) and (**b**) spiked eel muscle.

The retention times for the derivatized IDPs and amino acids were 17.9, 21.0, and 27.8 min for the β-, d-, and l-Ala derivatives, respectively; 37.1 and 39.9 min for the l- and d-His derivatives, respectively, and 37.9, 36.9, and 34.4 min for the Car, Ans, and Tau derivatives, respectively. The retention times for CIMa-Car, CIMa-Ans, and CIMa-Tau were comparable to that of CIMa-His (Figure 5a and Table 1). The difference between the retention times of CIMa-Car and CIMa-Ans was very small; however, this did not affect their detection by MS/MS. CIMa-Ans was eluted slightly earlier than CIMa-Car, and the same trend was observed when these IDPs were detected on a mixed-mode column without derivatization [28]. The derivatized IDP and amino acid peaks detected from the sashimi samples (Figure 5b, eel muscle) corresponded to those for the standard samples.

The pretreatment method in the present study differed slightly from that in our previous report [26]. Specifically, the derivatized samples were injected directly into the LC–MS/MS without solid-phase extraction (SPE). Nevertheless, good linearities (*R*^2^ > 0.999) were obtained for the derivatized IDPs and amino acids, even without SPE (Figure S2). Therefore, LC–MS/MS with (*R*)-CIMa-OSu derivatization can be applied to natural seafoods even if the concentrations of IDPs and amino acids vary.

The precision and accuracy of IDP and amino acid detection were subsequently examined by spiked experiments using eel homogenate, resulting in an intra-day accuracy of −13.9% to 4.12%, inter-day accuracy of −16.5% to 5.65%, intra-day precision of 0.60% to 13.2%, and inter-day precision of 0.40% to 13.9% (Table 1). These results demonstrate that both the accuracy and precision are satisfactory and that reliable measurements can be performed without interference from extraneous components in eels.

The LODs of the Car, Ans, and Tau derivatives were determined to be 5.73 × 10^−3^, 2.93 × 10^−2^, and 1.91 × 10^−2^ μg/mL, respectively, while for those for the d- and l-Ala and His derivatives ranged from of 1.22 × 10^−3^ to 8.96 × 10^−3^ μg/mL (Table 1). Comparing the LODs of the derivatized IDPs and amino acids, that of CIMa-Car was comparable to those of CIMa-Ala and CIMa-His, whereas the LOD of CIMa-Ans was approximately five times larger than that of CIMa-Car. Several reports indicate that Ans has a larger LOD than Car when measured by LC–MS/MS without derivatization [30,33], suggesting that the ionization efficiency of CIMa-Ans may be slightly lower with the present measurement conditions.

The trends in the magnitudes of the LODs of CIMa-Car and CIMa-Ans were very similar to those in other LC–MS/MS studies. Specifically, they were higher than those achieved by Pandya et al. by electrospray ionization (ESI)–MS/MS using a heated ESI probe [29]; comparable to those obtained by Uenoyama et al., Stautemas et al., and Peiretti et al. using ESI–MS/MS [28,30,34], and lower than those determined by Everaert et al. by ESI–MS/MS using a heated ESI probe [33] (Table 2). Moreover, the LOD was several orders of magnitude lower than those of other methods, including ultraviolet detection with different columns (e.g., with a C18 column [35,36], hydrophilic interaction LC column [37,38], Hypercarb^TM^ column [39], and amino column [40]) and fluorescence detection [41] (Table 2). These results suggest that the derivatization of Car and Ans with (*R*)-CIMa-OSu allows the simultaneous analysis of all relevant amino acids and Tau in seafoods, with comparative LODs to previous papers, even when only small amounts (<200 mg) of sample are available. The simultaneous measurement of IDPs and related amino acids, as well as Tau, which is generally abundant in seafoods, without SPE is particularly advantageous, because it significantly reduces the time required for pretreatment and analysis and the cost of using SPE cartridges. Thus, the present LC–MS/MS method can simultaneously analyze IDPs, related amino acids, and Tau using only a small fish sample.

### 3.4. Determination of Car, Ans, His, β-Ala, and Tau Concentrations in Natural Seafoods

**Figure 6:**
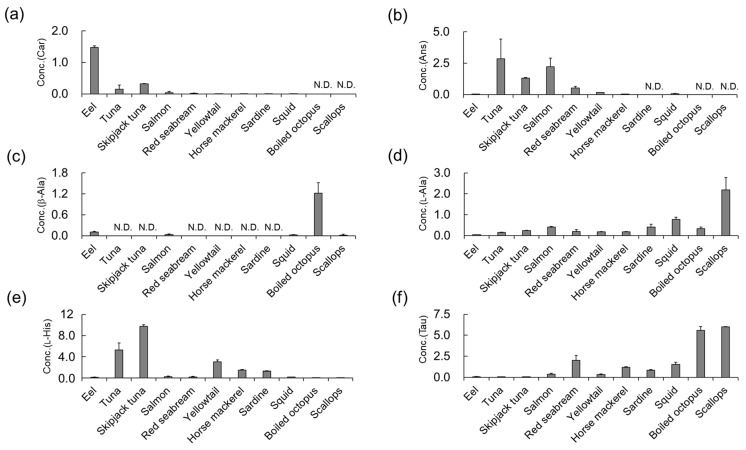
Concentrations of IDPs and amino acids in seafoods (mean ± SD [mmol/100 g-wet]): (**a**) Car, (**b**) Ans, (**c**) β-Ala, (**d**) l-Ala, (**e**) l-His, and (**f**) Tau.

The Car, Ans, l-Ala, l-His, and Tau contents in the seafood samples were determined using the developed LC–MS/MS approach with (*R*)-CIMa-OSu derivatization (Figure 6 and Table S5). Neither d-Ala nor d-His were detected in any sample. d-Ala is a selective and potentially co-agonist of the *N*-methyl-d-aspartate receptor and plays important roles in the brain [42]. Therefore, it has been studied in the field of psychiatric disorders. Since d-Ala is metabolized by d-amino acid oxidase (DAO), the combined administration of antipsychotic drugs and d-Ala has been reported to improve clinical symptoms in patients with schizophrenia [43]. Thus, the consumption of d-Ala is important. Representative foods with abundant d-amino acid contents are fermented foods (such as miso, yogurt, cheese, and black garlic) and vinegar [25,26,44]. d-Ala has been identified in aquatic invertebrates such as crustaceans (crabs and shrimp) and bivalves (clams and mussels); however, its concentration varies greatly among species [45]. Meanwhile, fish such as carp have been reported to contain DAO [46]. This suggests that both endogenous and exogenous d-Ala are metabolized and are therefore hardly detectable in the muscle tissue of fish. This could explain why d-Ala and d-His were not detected in the sashimi samples in this study.

As shown in Table S5 and Figure 6, the concentration of Car in the eel specimen (1.48 mmol/100 g-wet) (Figure 6a) was significantly higher than that in the other seafood samples (≤0.318 mmol/100 g-wet). Several previous studies have reported that eels, including conger eels [47] and sea eels [48], contain high concentrations of Car [39,47,49], with similar levels being detected to those in the current study. Car and Ans are reported to have anti-fatigue effects. A clinical study conducted by Shimizu et al. in 2009 showed that a daily intake of 400 mg of IDPs was effective in reducing fatigue within 2–8 weeks [50]. Eel, which had the highest concentration of Car in this study, provides 335 mg of Car per wet-100 g of eel muscle.

Since this study aimed to compare the concentrations of IDPs, related amino acids, and Tau in the muscles of seafoods, the eel sample was raw; however, unlike other sashimi such as tuna and salmon, eel is cooked (e.g., steamed or grilled) before eating. The concentrations of amino acids in seafoods change upon cooking; however, the extent and direction of change vary depending on the processing method and amino acids [51]. For example, the amino acid concentration typically increases upon cooking steamed patin catfish or steamed Spanish mackerel [52], but decreases upon cooking steamed shrimp [53], steamed oysters [54], or grilled mullet. Furthermore, there are no reports on how cooking changes the IDP contents of seafoods. In the future, it may be necessary to study the concentration of Car in eel muscle after cooking using our present method.

High concentrations of Ans were found in the tuna (2.84 mmol/100 g-wet), skipjack tuna (1.32 mmol/100 g-wet), and salmon (2.22 mmol/100 g-wet) specimens (Figure 6b). Similar trends have been reported previously [55,56], suggesting that IDPs may be required for long-distance swimming in these migratory pelagic fish. For tuna, which had the highest concentration of Ans in this study, six slices of sashimi (approximately 10 g each) would provide 400 mg of Ans. Although eating eel and tuna is a more efficient way to intake IDPs than eating other types of fish, these findings suggest that it may be difficult to achieve on a daily basis.

High levels of His were detected in the tuna (5.29 mmol/100 g-wet) and skipjack tuna (9.78 mmol/100 g-wet) samples (Table S5 and Figure 6e). This is consistent with reports that black marlin [57], yellowfin tuna [40,58,59], and skipjack tuna [47,57,59,60] contain high levels of His. Nimura et al., speculated that the high concentration of His in the muscles of tuna, which swim explosively, is due to the pH buffering capacity of His, which is inferior to that of IDPs [61]. This aligns with the high concentrations of His observed in the tuna and skipjack tuna specimens in the current study.

Finally, Tau was highly concentrated in the red seabream (2.01 mmol/100 g-wet), squid (1.53 mmol/100 g-wet), boiled octopus (5.59 mmol/100 g-wet), and scallop (6.02 mmol/100 g-wet) specimens (Figure 6f). These results are consistent with previous reports, which found high concentrations of Tau in squid [59] and octopus samples [62] and lower concentrations in fish, including horse mackerel and sardines [59]. However, Hiraoka et al., also reported that the Tau concentrations in red seabream were relatively low [59]. This may be because the Tau content varies widely between the white and red muscle in fish, with red muscle tending to have relatively high concentrations of Tau, possibly due to increased angiogenesis in these tissues [63].

Overall, the proposed method is promising for the comprehensive screening of IDPs and amino acids in foods, which will aid the consumption of these compounds by humans.

## 4. Conclusions

We developed an LC–MS/MS method based on pre-column derivatization with (*R*)-CIMa-OSu for the simultaneous determination of two IDPs, Car and Ans, in seafood samples. We also measured related amino acids and Tau using this approach. The method was validated for precision, accuracy, and sensitivity, and demonstrated robust performance. The IDP and amino acid concentrations in 11 kinds of natural seafoods were similar to those reported previously. This study demonstrates the effectiveness of (*R*)-CIMa-OSu for amino group derivatization, thereby enabling accurate LC–MS/MS determination of Car and Ans in seafoods. The method enhances our understanding of the nutritional content of seafoods and provides a valuable tool for future research into the health benefits and dietary significance of these compounds. Future studies could apply this method to different food matrices and investigate the biological implications of consuming seafoods rich in IDPs and Tau. In the future, (*R*)-CIMa-OSu could be employed to detect functional peptides in a variety of foods.

## Figures and Tables

**Table 1:** LOD, accuracy, and precision for quantification of derivatized IDPs and amino acids (*n* = 4).

Derivatized Component	RT ^1^ (min)	LOD (µg/mL)	Working Range (μM) Linearity (*R*^2^)	Spiked Concentration (μM)	Intra-Day (*n* = 4)		Inter-Day (*n* = 4)	
					Accuracy (RME ^2^, %)	Precision (RSD ^3^, %)	Accuracy (RME, %)	Precision (RSD, %)
Car	37.9	0.00573	6.25–500	20	−0.54	3.04	1.76	2.69
			0.99986	100	2.40	1.08	1.79	1.27
				200	4.12	1.69	3.36	0.40
Ans	36.9	0.0293	6.25–2000	50	−1.91	1.52	0.88	1.52
			0.99965	250	−2.38	0.80	−0.74	2.35
				500	−2.52	0.95	0.09	2.86
β-Ala	17.9	0.00122	6.25–200	10	−6.18	13.2	−3.31	13.9
			0.99961	50	−7.52	1.23	−4.52	3.32
				100	−2.99	2.09	−1.94	2.12
d-Ala	21.0	0.00194	6.25–200	10	−9.64	1.89	−8.58	3.83
			0.99962	50	−7.67	1.84	−5.93	2.95
				100	−6.69	1.31	−7.04	1.48
l-Ala	27.8	0.00201	6.25–1000	10	1.41	3.54	5.65	5.93
			0.99993	50	0.17	1.19	1.54	0.85
				100	−0.58	2.15	0.52	1.19
l-His	37.1	0.00826	6.25–3000	50	−1.78	1.51	−0.19	1.45
			0.99977	250	−4.55	0.60	−3.7	1.33
				500	−4.09	1.74	−4.53	1.32
d-His	39.9	0.00896	6.25–200	10	−13.01	2.66	−12.57	2.54
			0.99908	50	−11.19	1.16	−11.57	0.96
				100	−13.94	2.01	−15.25	1.78
Tau	34.4	0.0191	6.25–1000	20	−5.17	3.07	−6.56	10.8
			0.99994	100	−7.3	1.00	−13.75	9.60
				200	−7.75	1.07	−16.52	10.6

^1^ RT: retention time; ^2^ RME: relative mean error; ^3^ RSD: relative standard deviation.

**Table 2:** LODs of IDPs using different derivatization reagents, columns, and detection modes.

Derivatization Reagent	Column	Detection	LOD (µg/mL)		Ref.
			Car	Ans	
−	Mixed-mode	ESI–MS/MS	0.0044	0.0047	[28]
−	Peptide BEH C18	ESI–MS/MS with HESI ^2^ probe	0.000054	−	[29]
−	C18	ESI–MS/MS	0.0013	0.0026	[30]
−	C18	ESI–MS/MS with HESI probe	0.023	0.048	[33]
−	C18	ESI–MS/MS	0.011	0.011	[34]
Carbazole-9-carbonyl chloride	C18	UV (254 nm)	0.54	−	[35]
−	C18	UV (210 nm)	0.016	0.0096	[36]
−	HILIC	DAD ^1^ (214 nm)	5.6	8.2	[37]
−	Phosphorylcholine HILIC	UV (214 nm)	0.060	0.10	[38]
−	Hypercarb^TM^	UV (215 nm)	0.054	0.058	[39]
−	Amino	UV (210 nm)	0.042	0.039	[40]
*o*-Phthalaldehyde	Strong cation exchange	Fluorescence (ex. 340 nm, em. 445 nm)	0.14	0.22	[41]
(*R*)-CIMa-OSu	Mixed-mode	ESI–MS/MS	0.0057	0.029	This study

^1^ DAD: diode array detection; ^2^ HESI: heated electrospray ionization.

## Data Availability

The original contributions presented in the study are included in the article, further inquiries can be directed to the corresponding author.

## Supplementary Materials

The following supporting information can be downloaded at https://www.mdpi.com/article/10.3390/foods13121951/s1: Chemicals and Reagents, LC–MS/MS Analysis, Figures S1 and S2, Tables S1–S5. Figure S1: Negative collision-induced dissociation (CID) mass spectra and proposed mass fragmentation patterns of (a) CIMa-β-Ala and (b) CIMa-His; Figure S2: Calibration curves. The ratios of IDP and amino acid peak areas to the internal standard (IS) peak areas were plotted against the concentration of standard solution for (a) Car, (b) Ans, (c) β-Ala, (d) d-Ala, (e) l-Ala, (f) d-His, (g) l-His, and (h) Tau; Table S1: Multiple reaction monitoring conditions; Table S2: Weight per slice of sashimi used in this study (mean ± SD, *n* = 3); Table S3: Concentrations of calibration curves (μM); Table S4: Spiking concentrations (μM) added to eel homogenate; Table S5: Car, Ans, and amino acids contents of seafoods (mmol/100 g-wet, *n* = 3).
