# Supplementary material for: Determination of Imidazole Dipeptides and Related Amino Acids in Natural Seafoods by Liquid Chromatography–Tandem Mass Spectrometry Using a Pre-Column Derivatization Reagent
Source: Foods. 2024 Jun 20;13(12):1951. doi: 10.3390/foods13121951 (PMC11202704; doi:10.3390/foods13121951)
Supplement: Supplementary file 1 [file foods-13-01951-s001.zip › foods-3032645-supplementary.pdf]

Foods

Supporting Material

Determination of imidazole dipeptides and related amino acids in natural seafoods by liquid chromatography–tandem mass spectrometry using a pre-column derivatization reagent

Mayu Onozato, Minori Horinouchi, Yuki Yoshiba, Tatsuya Sakamoto, Hiroshi Sugasawa, Takeshi Fukushima

Department of Analytical Chemistry, Faculty of Pharmaceutical Sciences, Toho University, 2-2-1 Miyama, Funabashi-shi, Chiba 274-8510, Japan

Correspondence: Takeshi Fukushima, Ph.D.

Department of Analytical Chemistry, Faculty of Pharmaceutical Sciences, Toho University, 2-2-1 Miyama, Funabashi-shi, Chiba 274-8510, Japan

Tel./Fax: +81-47-472-1504; *E-mail:* t-fukushima@phar.toho-u.ac.jp

Contents

1. Supporting Figures

Fig. S1	Negative collision-induced dissociation (CID) mass spectra and proposed mass fragmentation patterns of (a) CIMa-β-Ala and (b) CIMa-His.	Page 3
Fig. S2	Calibration curves. The ratios of IDP and amino acid peak areas to the internal standard (IS) peak areas were plotted against the concentration of standard solution for (a) Car, (b) Ans, (c) β-Ala, (d) D-Ala, (e) L-Ala, (f) D-His, (g) L-His, and (h) Tau.	Page 4

2. Supporting Tables

Table S1	Multiple reaction monitoring conditions	Page 5
Table S2	Weight per slice of sashimi used in this study (mean ± SD, *n* = 3)	Page 5
Table S3	Concentrations of calibration curves (μM)	Page 6
Table S4	Spiking concentrations (μM) added to eel homogenate	Page 6
Table S5	Car, Ans, and amino acids contents of seafoods (mmol/100 g-wet, *n* = 3)	Page 7

1. Supporting Figures

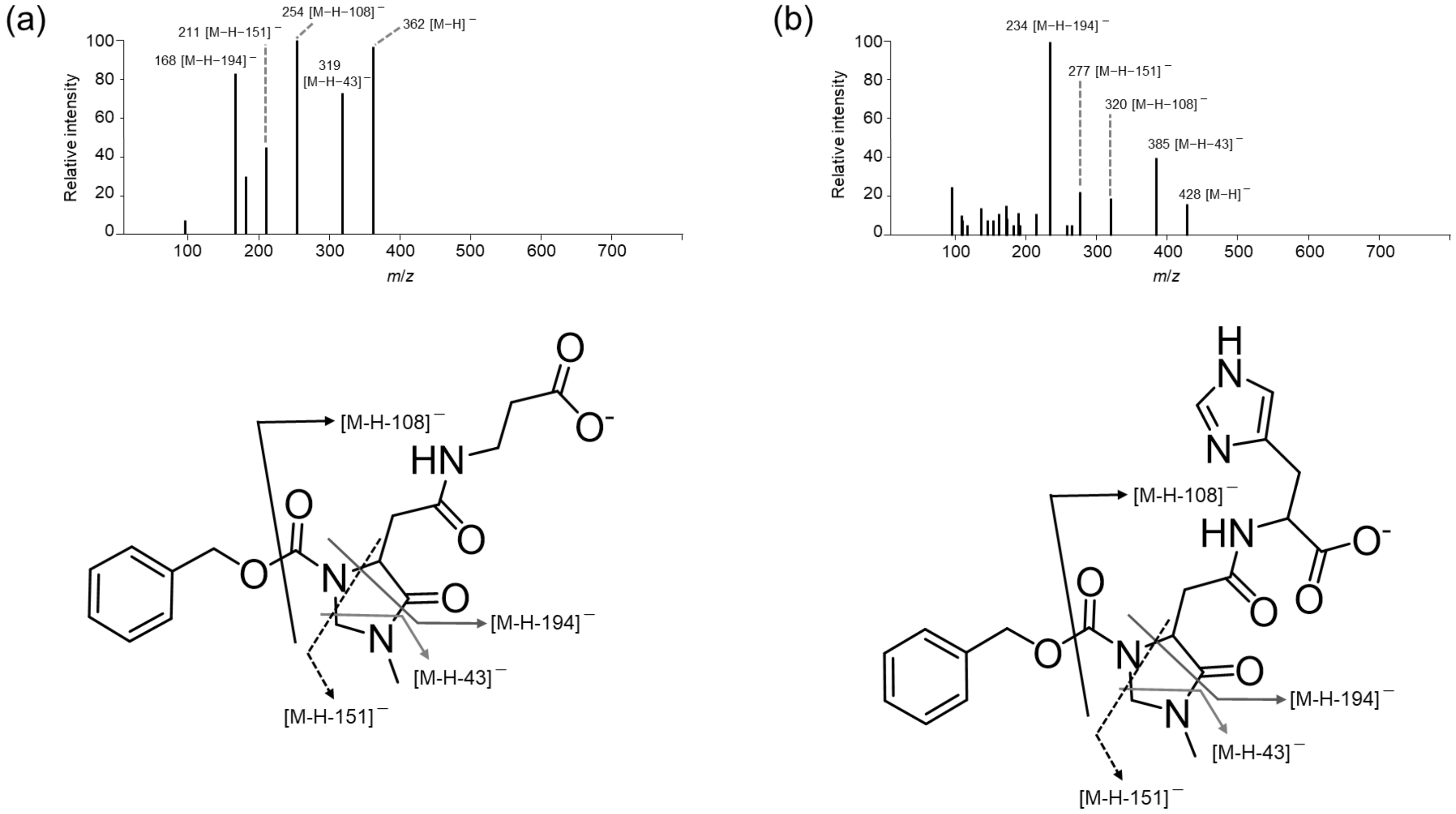

Fig. S1 Negative collision-induced dissociation (CID) mass spectra and proposed mass fragmentation patterns of (a) CIMa-β-Ala and (b) CIMa-His.

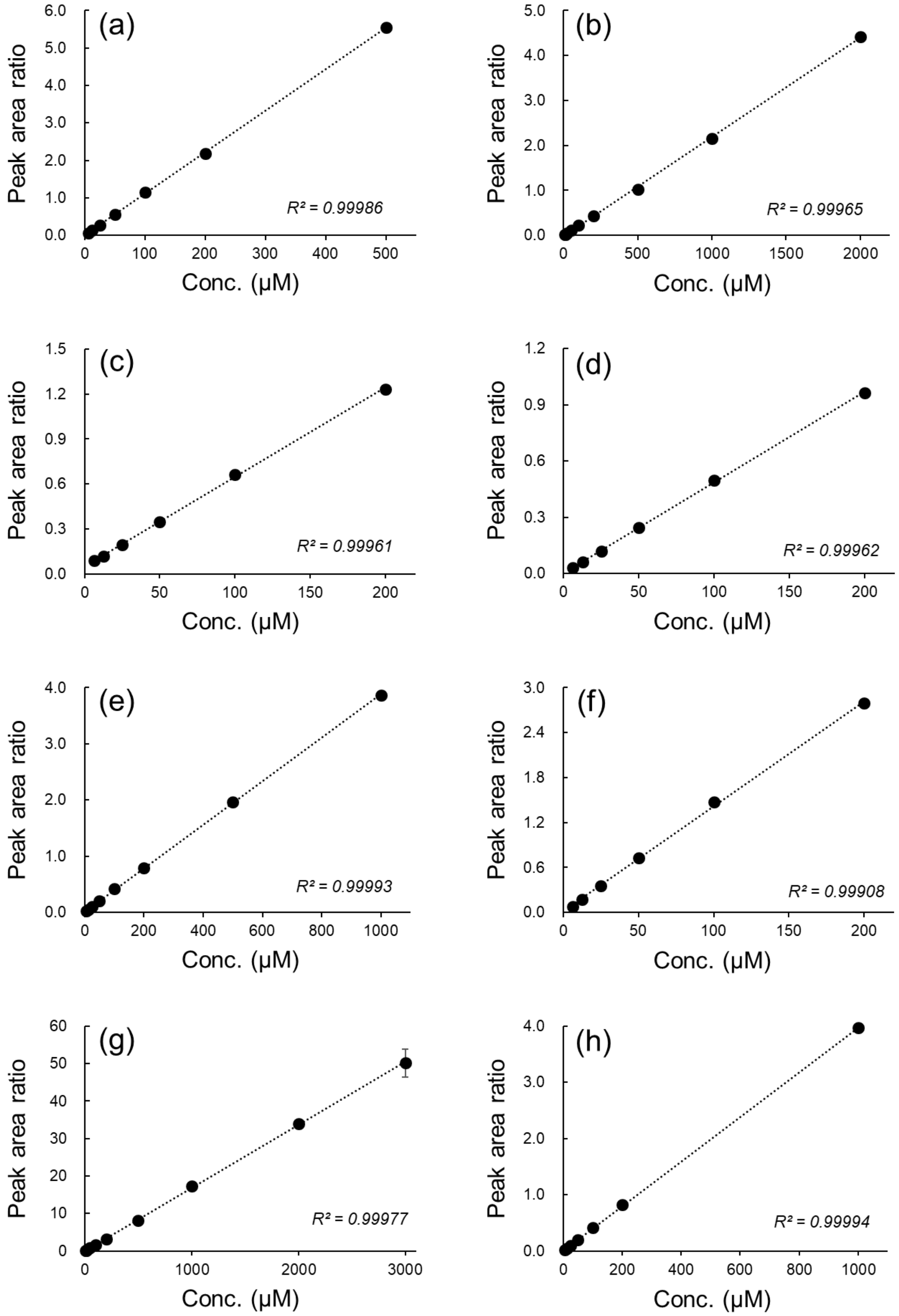

Fig. S2 Calibration curves. The ratios of imidazole dipeptide (IDP) and amino acid peak areas to the internal standard (IS) peak areas were plotted against the concentration of standard solution for (a) carnosine (Car), (b) anserine (Ans), (c) β-alanine (β-Ala), (d) D-alanine (D-Ala), (e) L-alanine (L-Ala), (f) D-histidine (D-His), (g) L-histidine (L-His), and (h) taurine (Tau).

2. Supporting Tables

Table S1 Multiple reaction monitoring (MRM) conditions

	m/*z*		Q1 Pre-Bias (V)	CE	Q3 Pre-Bias (V)
	Precursor	Product			
β-Ala	364.20	91.10	−24	−28	−18
D-Ala	364.20	91.10	−24	−28	−18
L-Ala	364.20	91.10	−24	−28	−18
Ala-IS[a]	367.20	91.10		−30	
L-His	430.20	91.10	−20	−35	−17
D-His	430.20	91.10	−20	−35	−17
His-IS[b]	439.20	91.10		−30	
Tau	400.05	91.10	−27	−30	−18
Car	501.20	91.10	−24	−51	−18
Ans	515.65	91.05	−28	−52	−19

[a]Ala-IS was used as an IS for β-Ala, D-Ala, and L-Ala.
[b]His-IS was used as an IS for L-His, D-His, Tau, Car, and Ans.

Table S2 Weight per slice of sashimi used in this study (mean ± SD, *n* = 3)

Sample	Weight (g)
Tuna	13.76 ± 2.40
Tuna	12.17 ± 0.71
Yellowfin tuna	8.81 ± 2.99
Skipjack tuna	19.14 ± 2.00
Salmon	12.36 ± 0.46
Salmon	10.95 ± 0.38
Salmon	10.85 ± 0.69
Red seabream	13.33 ± 1.64
Red seabream	8.50 ± 1.23
Yellowtail	12.20 ± 0.44
Horse mackerel	6.82 ± 0.63
Sardine	22.13 ± 0.56
Squid	1.78 ± 0.03
Scallops	8.29 ± 0.36
Scallops	14.36 ± 0.54

Table S3 Concentrations of calibration curves (μM)

Car	Ans	β-Ala	D-Ala	L-Ala	L-His	D-His	Tau
500	2000	200	200	1000	3000	200	1000
200	1000	100	100	500	2000	100	200
100	500	50	50	200	1000	50	100
50	200	25	25	100	500	25	50
25	100	12.5	12.5	50	200	12.5	25
12.5	50	6.25	6.25	25	100	6.25	12.5
6.25	25			12.5	50		6.25
	12.5			6.25	25		
	6.25				12.5		
					6.25		

Table S4 Spiking concentrations (μM) added to eel homogenate

Sample	Car	Ans	β-Ala	D-Ala	L-Ala	D-His	L-His	Tau
1	20	50	10	10	10	10	50	20
2	100	250	50	50	50	50	250	100
3	200	500	100	100	100	100	500	200

Table S5 Car, Ans, and amino acid contents of seafoods (mmol/100 g-wet, *n* = 3)

	Car	Ans	β-Ala	L-Ala	L-His	Tau
Eel	1.4793 ± 0.0508	0.0433 ± 0.0046	0.1050 ± 0.0225	0.0390 ± 0.0033	0.1343 ± 0.0256	0.0780 ± 0.0324
Tuna	0.0053 ± 0.0004	4.6727 ± 0.7028	N.D.	0.1674 ± 0.0119	3.8454 ± 0.4840	0.0636 ± 0.0218
Tuna	0.2701 ± 0.0126	1.8216 ± 0.1505	N.D.	0.1395 ± 0.0095	6.3257 ± 0.2273	0.0684 ± 0.0082
Yellowfin tuna	0.1556 ± 0.0069	2.0293 ± 0.4174	N.D.	0.1543 ± 0.0079	5.7036 ± 0.3103	0.0637 ± 0.0110
Skipjack tuna	0.3185 ± 0.0163	1.3177 ± 0.0304	N.D.	0.2493 ± 0.0098	9.7813 ± 0.2703	0.0730 ± 0.0133
Salmon	0.0179 ± 0.0023	2.2361 ± 0.1930	0.0081 ± 0.0008	0.3918 ± 0.0168	0.2870 ± 0.0418	0.3088 ± 0.0647
Salmon	0.0821 ± 0.0028	1.5324 ± 0.0633	0.0356 ± 0.0046	0.4425 ± 0.0235	0.3126 ± 0.0124	0.3119 ± 0.0761
Salmon	0.0017 ± 0.0012	2.8850 ± 0.0554	0.0460 ± 0.0017	0.3897 ± 0.0647	0.1277 ± 0.0549	0.5191 ± 0.0961
Red seabream	0.0090 ± 0.0012	0.6158 ± 0.0148	N.D.	0.2616 ± 0.0179	0.2717 ± 0.0390	2.4226 ± 0.2872
Red seabream	0.0195 ± 0.0036	0.4056 ± 0.0582	N.D.	0.1310 ± 0.0274	0.0564 ± 0.0164	1.6037 ± 0.4730
Yellowtail	0.0032 ± 0.0002	0.1624 ± 0.0071	N.D.	0.1888 ± 0.0128	3.0456 ± 0.3189	0.3269 ± 0.0853
Horse mackerel	0.0038 ± 0.0010	0.0459 ± 0.0027	N.D.	0.1885 ± 0.0110	1.4709 ± 0.0732	1.1695 ± 0.0662
Sardine	0.0006 ± 0.0001	N.D.	N.D.	0.4133 ± 0.1320	1.2733 ± 0.0783	0.8253 ± 0.0871
Squid	0.0034 ± 0.0015	0.0711 ± 0.0126	0.0251 ± 0.0062	0.7683 ± 0.1157	0.1414 ± 0.0471	1.5252 ± 0.2681
Boiled octopus	N.D.	N.D.	1.2189 ± 0.2969	0.3408 ± 0.0638	0.0216 ± 0.0028	5.5895 ± 0.4541
Scallops	N.D.	N.D.	0.0374 ± 0.0029	2.6042 ± 0.1097	0.0741 ± 0.0019	5.9931 ± 0.5388
Scallops	N.D.	N.D.	N.D.	1.7756 ± 0.2384	0.0701 ± 0.0052	6.0422 ± 0.6526

Tuna, salmon, red seabream, and scallops were purchased from two or three different local supermarkets.
